# Differential expression of *BnSRK2D* gene in two *Brassica napus* cultivars under water deficit stress

**Published:** 2014-12

**Authors:** Bahlanes Bakhtari, Hooman Razi

**Affiliations:** Department of Crop Production and Plant Breeding, College of Agriculture, Shiraz University, Shiraz, Iran

**Keywords:** Rapeseed, SnRK2, BnABF2, Drought tolerance, Transcript accumulation

## Abstract

The sucrose non-fermenting 1-related protein kinase 2 (SnRK2) family members are plant unique serine/threonine kinases which play a key role in cellular signaling in response to abiotic stresses. The three SnRK2 members including SRK2D, SRK2I and SRK2E are known to phosphorylate major abscisic acid (ABA) responsive transcription factors, ABF2 and ABF4, involved in an ABA-dependent stress signaling pathway in Arabidopsis. This study aimed to clone and sequence an ortholog of the Arabidopsis *SRK2D* gene from *Brassica napus*, designated as *BnSRK2D*. An 833bp cDNA fragment of *BnSRK2D*, which shared high amino acid sequence identity with its Arabidopsis counterpart, was obtained suggesting a possible conserved function for these genes. The expression pattern of *BnSRK2D* and its potential target gene *B. napus* ABF2 (*BnABF2*) were then analyzed in the two cultivars with contrasting reaction to water deficit stress. Semi-quantitative reverse transcription polymerase chain reaction (RT-PCR) showed that *BnSRK2D* and *BnABF2* were water-deficit stress responsive genes with similar expression profiles. The accumulation of the *BnSRK2D* and *BnABF2* transcripts in the two cultivars was linked with their level of drought tolerance, as the drought tolerant cultivar had significantly higher expression levels of both genes under normal and water deficit stress conditions. These findings suggest that *BnSRK2D* and *BnABF2* genes may be involved in conferring drought tolerance in *B. napus*.

## INTRODUCTION

Plants are frequently confronted with various abiotic stresses such as extreme temperatures, high salinity and drought that adversely affect their growth and productivity. To sense and respond to environmental stresses, plants have developed a series of molecular and physiological mechanisms. Stress signals are perceived by and transduced to different cellular compartments by a complex signaling network in which reversible protein phosphorylation catalyzed by protein kinases and protein phosphatases is a key component of the cellular regulatory apparatus [1, 2]. Genetic studies have revealed that several stress-inducible protein kinases are activated by Abscisic acid (ABA) and diverse stress signals. For instance, plant mitogen-activated protein kinases (MAPKs) are involved in stress signal transductions [3-5]. Calcium dependent protein kinases (CDPKs) are other major components of signaling pathways responding to abiotic stresses such as salinity and drought [6, 7]. The plant SNF1-related protein kinase (SnRK) family, which belongs to the CDPK-SnRK superfamily [8], has been identified as a key regulator in linking stress and metabolic pathways [9, 10]. 

Plant SnRKs are classified into three subfamilies: SnRK1, SnRK2 and SnRK3 [8, 11]. Evidence shows that SnRK1 kinases have a role in the regulation of carbon and nitrogen metabolism [12]. SnRK2 and SnRK3 subfamilies are plant specific serine-threonine kinases which mainly act in signaling stress [8]. SnRK2s are a cross-talk point of ABA-dependent and –independent pathways for osmotic stress responses [13]. The first SnRK2 cDNA clone, *PKABA1*, was discovered from an ABA-treated wheat embryo cDNA library [14]. Another member of the SnRK2 subfamily, designated AAPK (ABA activated protein kinase), was later identified as a central regulator of ABA-dependent stomatal closure in fava beans [15, 16]. The SnRK2 family has ten members (SnRK2.1 to -2.10/SRK2A to –J) in the Arabidopsis genome [17], all of which are activated by hyperosmotic and salinity stresses (except for SnRK2.9/SRK2J), five are activated by ABA (SnRK2.2/SRK2D, SnRK2.3/SRK2I, SnRK2.6/SRK2E, SnRK2.7/SRK2F and SnRK2.8/SRK2C), and none upregulated by cold stress [17-19]. Furthermore, SnRK2.8/SRK2C over-expression caused the upregulation of stress related genes and led to the improvement of drought tolerance in Arabidopsis [20]. SnRK2.4 and SnRK2.10 are known to be involved in root growth and architecture in saline conditions [21]. The three phylogenetically close SnRK2 members (SnRK2.2/ SRK2D, SnRK2.3/SRK2I, SnRK2.6/SRK2E) are known to function as key regulators of ABA signaling in response to osmotic stress [13, 22, 23] and control seed development and dormancy [24]. These three members phosphorylate the ABA-responsive transcription factors, ABF2 and ABF4, [25] which play major roles in an ABA-dependent stress signaling pathway [26, 27]. Members of the SnRK2 family have been identified in a number of crop plants including rice [28], maize [29], wheat [30, 31], soybean [32, 33] and tobacco [34], and were shown to be activated under single or multiple environmental stresses [28-30, 32]. Moreover, the over-expression of some SnRK2 orthologs such as *TaSnRK2.4* [35] and *TaSnRK2.8* [36] in wheat, *SAPK4* in rice [37] and *ZmSPK1* in maize [38] enhanced abiotic stress tolerance. These findings reveal the critical functions of SnRK2 genes in mediating osmotic stress signaling and tolerance; however whether each SnRK2 gene shows significant variation in expression patterns between different genotypes under various abiotic stresses or not remains to be understood. 

 As one of the world’s leading oilseed crops,* Brassica napus* (rapeseed), is substantially affected by adverse environmental factors. Knowledge of the molecular basis of its response mechanisms to abiotic stresses is, therefore, essential for transgenic breeding and improved stress tolerance. In the present study, we cloned, sequenced and characterized the partial cDNA fragment of the *B. napus* *SRK2D* (SnRK2.2;* BnSRK2D*) gene for the first time. We also demonstrated the expression of *BnSRK2D* transcripts and its potential target gene *BnABF2* in response to water deficit stress.

## MATERIALS AND METHODS

**Plant materials and water stress treatment: **Two winter- type *B. napus* cultivars, Karaj1 (originated from Iran) and SLM046 (originated from Germany), were used in this study. Seeds were obtained from the Seed and Plant Improvement Institute, Karaj, Iran. The Karaj1 cultivar has been shown to be more drought tolerant than SLM046 [39]. The plants were grown in plastic pots filled with sterilized soil under greenhouse conditions with 16 hours of daylight and a 25ºC day temperature. Soil water content at field capacity (FC) was 25% (w/w). Leaf samples were collected separately from three-week old plants under three different soil moisture conditions including non-stress (control), water deficit stress and re-watering. In the control treatment, plants were regularly irrigated to maintain soil moisture at the FC level (100% FC). The water deficit treatment was imposed on the plants by withholding water to reduce soil moisture to 50% of the FC. Subsequently, the recovery of the water stressed plants after re-watering was examined for the genes of interest.

**Cloning and sequencing of** ***BnSRK2D*** **partial cDNA: **Total RNA was isolated from the leaves of the SLM046 cultivar using a DENAzist column RNA isolation kit according to the manufacturer’s protocol. The extracted RNA was then treated with DNase I (Fermentas) according to the manufacturer’s instruction to remove genomic DNA contamination. RNA quantity and quality was determined by agarose gel electrophoresis and spectrophotometry (Nanodrop). First strand cDNAs were synthesized from DNase-treated RNA using Viva 2-steps RT-PCR kit (Vivantis). Since *B. napus* and *A. thaliana* share a high level of similarity in coding regions, the specific primers, AtSRK2D forward (5΄-CCG ATA GAG TCA CCA AGG AGC-3΄) and AtSRK2D reverse (5΄-CGT AGC CTC CGA TAT TAT CTG C-3´) were designed from the exon regions of the *A. thaliana* *SRK2D* gene (*AtSRK2D*; GenBank accession number: NM-114910). The PCR reaction mixture (20 µl) consisted of 1µl of the first strand cDNA, 0.25 mM of each dNTP, 0.4 mM of each primer, 2mM of MgCl2, 1x PCR buffer and 1U Taq DNA polymerase. The PCR reaction was carried out as follows: initial denaturation at 94°C for 2.5 min followed by 30 cycles of 94°C for 30 sec, 58°C for 30 sec, 72°C for 1 min, and a final extension at 72°C for 5 min. The PCR product was analyzed by agarose gel electrophoresis (1% w/v). Thereafter, the obtained PCR product was purified and cloned into the pTZ57R/T vector (Fermentas) and sequenced. Sequencing was performed by Bioneer (Korea) using standard M13 forward and M13 reverse primers.

**DNA and protein sequence analysis:** Sequence similarity was searched using the BLAST program (http://www.ncbi.nlm.nih.gov/blast). To identify the putative SRK2D ortholog in *Brassica rapa* (*BrSRK2D*), a BLAST homology search was performed using the sequenced fragment of *BnSRK2D* against *B. rapa* genome in Phytozome (www.phytozome.net). The deduced amino acid sequence of the genes and multiple alignment of protein sequences were obtained using Vector NTI software. A functional domain was identified within Brassica SRK2D proteins by Pfam (pfam.sanger.ac.uk). Based on amino acid sequences of the Arabidopsis and Brassica SRK2 genes, the phylogenetic tree was constructed by Mega version 5 program using a neighbor-joining method with 500 bootstrap replicates.

**Expression analysis of** ***BnSRK2D*** **and** ***BnABF2*** **genes: **Semi-quantitative RT-PCR was performed to analyze expression levels of *BnSRK2D* and its downstream potential target gene, *BnABF2*, under various moisture regimes. Total RNA was extracted from the leaves of SLM046 and Karaj1 cultivars in non-stress, water deficit stress and re-watering conditions using a DENAzist column RNA isolation kit. The cDNAs consisted of equal amounts of DNase-treated total RNA samples using a Viva 2-steps RT-PCR kit (Vivantis) following the manufacturer’s instruction. The cDNAs were amplified for 28 cycles using the forward primer, BnSRK2DF (5΄-AAC CTC TCA CCA GGA TGT CGC-3΄), and the reverse primer, BnSRK2DR (5΄-CGT AGC CTC CGA TAT TAT CTG C-3΄), designed from the sequenced fragment of *BnSRK2D* and BnABF2R (5΄-TCC AGC AAG TGG AAT AAC ACC-3΄) and BnABF2F (5΄-GGA ATG AGC CAC CAG GAG ATG G-3΄) designed from *BnABF2* (accession number: HE 616527). RT-PCR reactions were also performed for the reference gene, *B. napus* *Elongation Factor1* (*BnEF1*; accession number: FJ529181), using the specific primers BnEF1F (5΄-AGC CGC AAG TCC TCC TCT CAG-3΄) and BnEF1R (5΄-TTC ATC TCA GCA GCC TCC TTC TCG-3΄). RT-PCR products were separated on 1% agarose gel. Amplicon band intensities were quantified using the Total Lab software. The expression levels of *BnSRK2D* and *BnABF2* were normalized relative to the amount of *BnEF1* expression. Normalized expression values of *BnSRK2D* and *BnABF2* were presented as fold change over the respective control (the expression of the genes of interest in the cultivar, SLM046 under normal conditions). 

The experiment was independently repeated three times. A one-way analysis of variance was conducted to evaluate *BnSRK2D* and *BnABF2* expressions between the cultivars under the three different moisture conditions. Mean values were compared by Tukey’s test (P<0.05). A correlation coefficient was calculated between the expression data of *BnSRK2D* and *BnABF2*.

## RESULTS AND DISCUSSION

**Identification and sequence analysis of Brassica** ***SRK2D*** **genes: **Using specific primers designed from *AtSRK2D*, RT-PCR successfully amplified a cDNA fragment from *B. napus* cultivar, SLM046, with the expected size. Cloning followed by sequencing of the isolated cDNA fragment revealed an 833bp *B. napus* *SRK2D* gene, designated as *BnSRK2D*. The cDNA sequence was submitted to the GenBank under the accession number LK937699. *BnSRK2D* showed high nucleotide homology with *AtSRK2D* (88%) and *AtSRK2I* (84%). The genomes of Arabidopsis and Brassica species belong to the same family (Brassicaceae), and their genomes share extensive similarities providing a solid foundation for comparative genomics. We also obtained the full coding sequence (1086 bp) of the *B. rapa* SRK2D (*BrSRK2D*; Phytozome accession number: Brara.C04319.1) ortholog by searching the Phytozome database. *B. rapa* is one of the diploid progenitors of the tetraploid *B. napus* [40]. The fully sequenced genome of *B. rapa* serves as an important resource to strengthen research on the evolution of polyploid genomes as well as the genetic improvement across the cultivated Brassica species [41].* BrSRK2D* showed 97% and 88% nucleotide identity to *BnSRK2D* and *AtSRK2D*, respectively.

**Figure 1:**
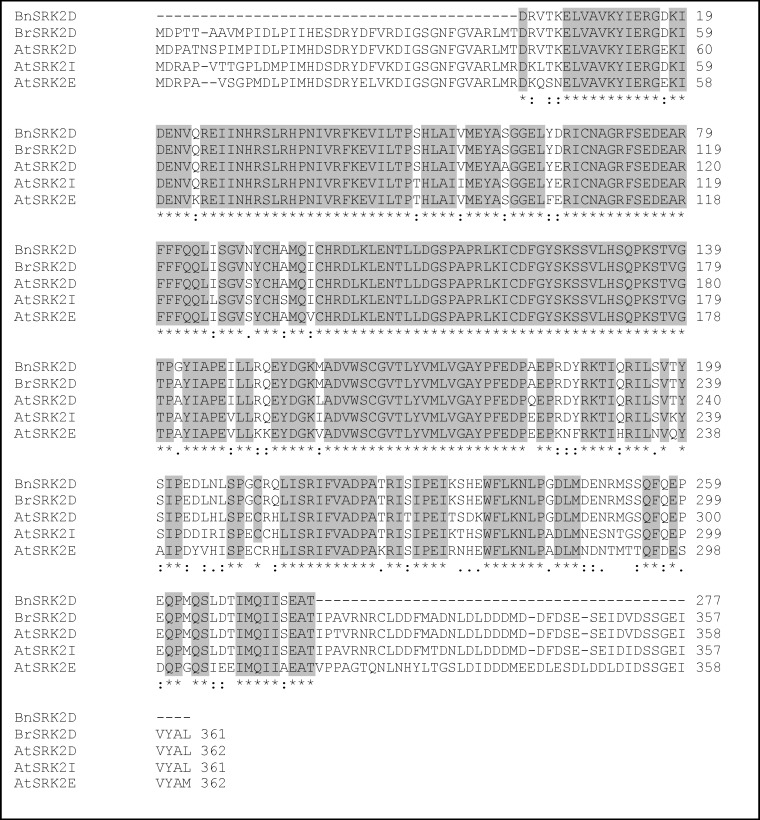
Alignment of the amino acid sequences of Arabidopsis SRK2 subgroup 3 including AtSRK2D (NP-190619), AtSRK2I (NP-201489), AtSRK2E (NP-567945) as well as *B. napus* SRK2D (BnSRK2D; CDU84836) and *B. rapa* SRK2D (BrSRK2D; Phytozome accession number: Brara.C04319.1). (*), (:) and (.) represent identical, conserved and semi conserved residues, respectively

The *BnSRK2D* fragment deduced protein consisted of 277 amino acid residues with the entire open reading frame of *BrSRK2D* encoding a protein of 361 amino acid residues. As Figure 1 shows, the multiple amino acid alignment revealed that *BnSRK2D* and *BrSRK2D* had significant sequence similarity with the members of the third subgroup of Arabidopsis SRK2 genes (*AtSRK2D*, *AtSRK2I* and *AtSRK2E*). Based on phylogeny, the Arabidopsis SRK2 family members are divided into three subgroups which differ in their activation in response to ABA [28]. Subgroup 1 members are not activated by ABA (*AtSRK2J*, *AtSRK2G*, *AtSRK2H*, *AtSRK2A* and *AtSRK2B*), while the members of subgroup 2 (*AtSRK2C* and *AtSRK2F*) are weakly activated in response to ABA [2]. The members of subgroup 3 are strongly activated by ABA and have key roles in ABA signal transduction pathways [13, 17, 24]. *BnSRK2D* shares 99% and 95% amino acid identities with *BrSRK2D* and *AtSRK2D*, respectively, suggesting their conserved function with regards to the phosphorylation of target proteins. This was supported by evidence showing the existence of a protein kinase domain within the proteins of *BnSRK2D* (from 1^st^ to 238^th^ amino acid residues) and *BrSRK2D *(from 22^nd^ to 278^th^ amino acid residues).

**Figure 2:**
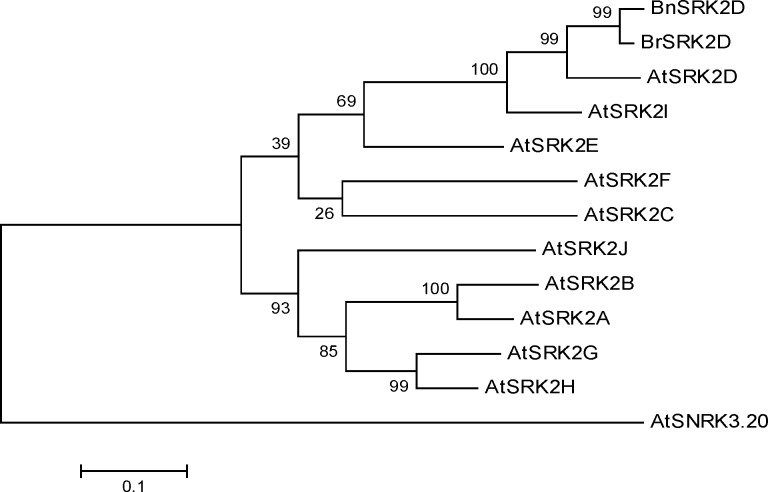
Phylogenetic tree of the amino acid sequences of Arabidopsis SRK2s [AtSRK2A (NP-172563), AtSRK2B (NP-849834), AtSRK2C (NP-974170), AtSRK2D (NP-190619), AtSRK2E (NP-567945), AtSRK2F (NP-195711), AtSRK2G (NP-196476), AtSRK2H (NP-201170), AtSRK2I (NP-201489), AtSRK2J (NP-179885)], *Brassica napus* SRK2D (BnSRK2D; CDU84836) and *Brassica rapa* SRK2D (BrSRK2D; Phytozome accession number: Brara.C04319.1). Arabidopsis SRK3.20 (AtSRK3.20; NP-174217) was used as the outgroup. The numbers at the nodes are bootstrap values

In order to infer phylogenetic relationships between Brassica *SRK2* genes and their counterparts in the close relative species, *Arabidopsis thaliana*, the phylogenetic tree was reconstructed using aligned amino acid sequences (Fig. 2). The tree showed that Arabidopsis and Brassica SRK2s were grouped together but separated from AtSnRK3.20 as the outgroup. Similar to previous studies, Arabidopsis SRK2s were classified into three subgroups [28]. Brassica SRK2s fell within subgroup 3 as orthologs of *AtSRK2D* supported by a high bootstrap value. The presence of *SRK2D* genes from diploid *B. rapa* and tetraploid *B. napus* within the same clade supported the view that gene duplication occurred before the divergence of the diploid Brassica species [40].

**Figure 3:**
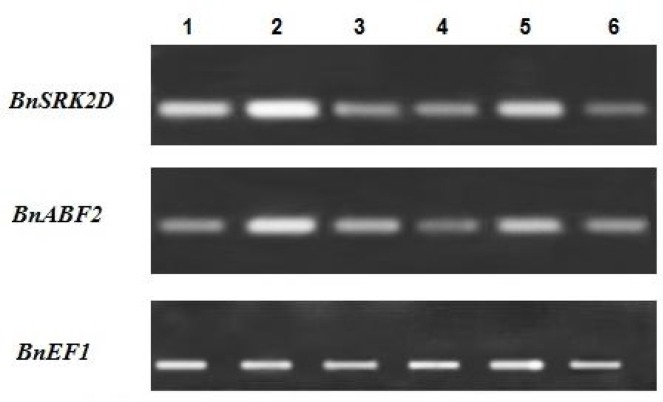
Expression of *BnSRK2D* and *BnABF2* genes in leaves of the two *B. napus* cultivars under different water conditions. Lanes 1 to 3 are the RT-PCR products obtained from non-stress, water deficit stress and rewatering conditions, respectively for the drought-tolerant *B. napus* cultivar (Karaj 1). Lanes 4 to 6 are the RT-PCR products obtained from non-stress, water deficit stress and re-watering conditions, respectively for the drought-sensitive *B. napus* cultivar (SLM046). *BnEF1* was used as the control gene

**Expression analysis of** ***BnSRK2D*** **and** ***BnABF2*** **genes: **In order to examine the expression pattern of *BnSRK2D* and *BnABF2* genes under water deficit stress, semi-quantitative RT-PCR analyses were performed. RT-PCR specifically amplified 216bp and 212bp cDNA fragments of *BnSRK2D* and *BnABF2*, respectively (Fig. 3). 

The highly significant correlation coefficient (0.91) between the expression data of these genes revealed that the expression patterns of *BnSRK2D* and *BnABF2* were in accordance with each other. The involvement of Arabidopsis *SRK2D* and *ABF2* genes in the same stress signaling pathway has been previously reported as ABF2 is the target transcription factor to be phosphorylated by SRK2D [25]. 

**Figure 4:**
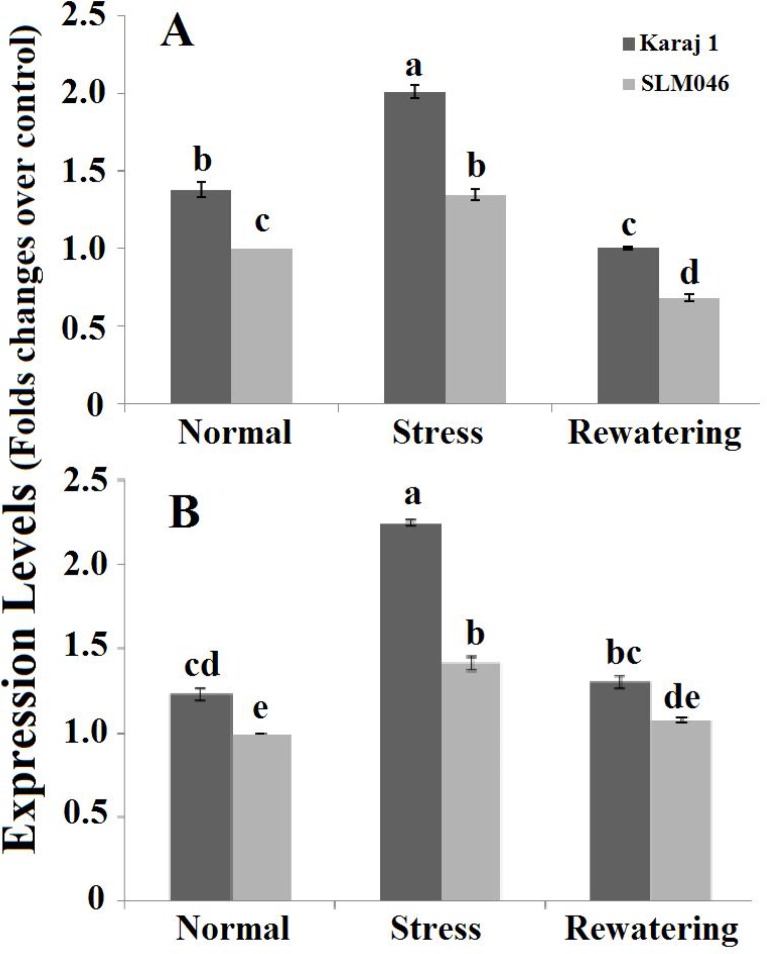
Comparison of the expression levels of *BnSRK2D* (A) and *BnABF2* (B) under normal, water deficit stress and re-watering conditions in the two *B. napus* cultivars, Karaj 1 and SLM046. The expression data were normalized to *BnEF1* expression and shown as fold change over the respective control (the expression of *BnSRK2D* and *BnABF2* genes in cultivar SLM046 under normal conditions). Data represent means ± SE from three independent experiments. The means with the same letter are not significantly different (P<0.05) according to Tukey’s test

The cultivars also showed similar trends for the expressions of *BnSRK2D* and *BnABF2*, although the drought tolerant cultivar (Karaj1) had significantly higher expression levels of both genes under all water conditions (Fig. 4). The expressions of *BnSRK2D* and *BnABF2* were significantly upregulated in response to water deficit stress; however, the increase was higher in the drought tolerant cultivar. Moreover, similar to their Arabidopsis counterparts, a significant reduction of the transcripts of *BnSRK2D* and *BnABF2* genes was observed under re-watering treatment, confirming the responsiveness of the genes to osmotic stress [17, 27]. Together, the accumulation of *BnSRK2D* and *BnABF2* transcripts in the two cultivars was associated with their level of drought tolerance. In conclusion, the findings suggest that *BnSRK2D* and *BnABF2* genes may be involved in conferring drought tolerance in *B. napus*. 

This is the first report on the cloning and expression analysis of *BnSRK2D* aimed at elucidating its function in response to water deficit stress. Further in-depth analyses on the functions of *B. napus* SRK2s are needed to fully understand the importance of these genes in stress signaling pathways and their subsequent transgenic breeding aimed at improving drought tolerance.

## Notes

**Acknowledgement: **The authors would like to thank Shiraz University for funding this research.
